# Efficacy of mesalazine in children with moderate to severe alopecia areata: case series of 18 patients^☆^

**DOI:** 10.1016/j.abd.2023.02.002

**Published:** 2023-07-03

**Authors:** Analú Vivian, Vania Oliveira de Carvalho, Ana Elisa Kiszewski

**Affiliations:** a Service of Dermatology, Universidade Federal de Ciências da Saúde de Porto Alegre, Porto Alegre, RS, Brazil; b Department of Pediatrics, Universidade Federal do Paraná, Curitiba, PR, Brazil; c Department of Internal Medicine, Universidade Federal de Ciências da Saúde de Porto Alegre, Porto Alegre, RS, Brazil; d Pediatric Dermatology Unit, Hospital da Criança Santo Antônio, Irmandade Santa Casa de Misericórdia de Porto Alegre, Porto Alegre, RS, Brazil

Dear Editor,

Alopecia areata (AA) mainly affects the scalp but can also affect eyelashes and body hair. The treatment can be topical (corticosteroids, minoxidil, anthralin, diphencyprone) or systemic (corticosteroids, azathioprine, methotrexate, cyclosporine, and sulfasalazine). ^1^ Studies have demonstrated the benefit of using mesalazine^2^ and, more recently, Janus kinase (JAK) inhibitors.^3^

Up to June 2022, the FDA had not approved any treatments for AA, at which time it approved baricitinib for adults.^4^ Until then, treatment options were off-label^2^ and JAK inhibitors constitute medications that remain difficult to access, due to their high cost. Patients with severe disease show limited benefit from treatments and the recurrence rate is high. Therefore, the risk/benefit ratio and safety of the drugs used in the treatment must be considered. AA affects both adults and children, and it is estimated that up to 20% of cases occur in childhood,^5^, ^6^ when it tends to be more severe and show a worse prognosis.^3^ Children with AA have higher rates of anxiety, depression, and decreased quality of life.^7^

This is a study of 18 patients carried out in two units of Pediatric Dermatology in Brazil, one at Hospital Santo Antônio, in Porto Alegre, and the other at Hospital das Clínicas, in Curitiba. The study period, from the selection phase to the final evaluation, was from January 2019 to July 2020. Of the 18 patients, two did not respond to mesalazine after six months of treatment. Two others were unable to complete their follow-up and treatment due to their moving to another city or due to the Coronavirus-19 pandemic. Thus, 14 patients were analyzed at six and 12 months after starting treatment: ten with AA in plaques with a Severity of Alopecia Tool (SALT) score >40, universal alopecia (UA) or total alopecia (TA), and four with diffuse AA. Of these four, three had the diagnosis confirmed by histopathology, and one initially had AA in plaques, but progressed to diffuse AA. Individuals with a diagnosis of AA with a SALT score ≥40 or with diffuse AA, unresponsive to topical treatment and systemic corticosteroid therapy, of either sex, aged between two and 18 years were included. Informed consent was obtained from all patients and/or their legal guardians.

Laboratory tests were performed before treatment, monthly in the first six months and every two months until completing 12 months. During treatment with mesalazine, patients with a positive hair pull test (indicating active disease) used systemic corticosteroid therapy: prednisolone 1 mg/kg/day for five days, with gradual withdrawal from the sixth day onward, for 22 to 30 days. Weekend maintenance was performed at a dosage of 0.5 to 1 mg/kg/day until the hair pull test was negative. All patients in the study had received therapy with systemic corticosteroids alone at the same dosages as described above for three months before initiating mesalazine and showed no signs of repilation. Mesalazine was administered orally, at an initial dosage of 30 mg/kg/day, divided into two daily dosages, which were increased up to 50 mg/kg/day.

Treatment response was assessed using photographs applying the SALT score.^6^ The score ranges from zero (absence of alopecia) to 100 (total absence of hair on the scalp). Treatment response was assessed at six and 12 months of therapy by the difference in the baseline SALT score (SALT B) and the scores at these two times (SALT 6 months; SALT 12 months). The patients were classified into four groups according to the percentage of reduction in the SALT score: 5% to 25% (weak response), 26% to 50% (moderate response), 51% to 75% (significant response), and 76% to 100 % (highly significant response).

To calculate treatment efficacy, comparisons of the SALT scores obtained using Student *t* test were performed. Patients with diffuse AA could not be assessed using the SALT score because it refers to plaque assessment. Therefore, follow-up and improvement were assessed through photographs (at baseline, six and 12 months) and through dermoscopy, by looking for black dots, exclamation point hairs, and yellow dots. The group with diffuse alopecia was classified as follows: excellent when repilation was assessed by clinical examination and dermoscopy was > 40%, good when it was 20%‒40%, poor when < 10%, and no response when there was no change. The results of laboratory tests obtained before and after the use of mesalazine showed no changes. The baseline SALT score ranged from 40.6 to 100, with a mean of 86.5 (SD = 22.2). The results indicate significant efficacy of the treatment with mesalazine, both at six and 12 months (Table 1). The mean relative reduction at six months was 29.9%, p = 0.003; (range: -67; 0; n = 12) and at 12 months it was 66.8%, p < 0.001; (range: -92.2; -12.0; n = 10). After 12 months of therapy with mesalazine, 40% of the patients showed a highly significant response (reduction ≥ 76%) – Fig. 1; 30% showed a significant response, 20% a moderate response and one (10% of the sample) showed low response to the medication (Table 2). The four patients with diffuse AA showed significant improvement and achieved an excellent response after 12 months of treatment (Table 2).

**Table 1:** Patients outcomes at six and 12 months after mesalazine treatment and score range

Six months – basal change (n = 12)	Basal	Six months	Six months – basal change
Mean ± SD	86.5 ± 22.2	60.6 ± 29.8	−25.8^a^ ± 23.4
Range	40.6‒100	15.2; 100	−67.2; 0
Mean reduction (%)			−29.9

Twelve months – basal change (n = 10)	Basal	Twelve months	Twelve months – basal change
Mean ± SD	83.8 ± 23.6	27.8 ± 25.7	−56.0^b^ ± 28.4
Range	40.6; 100	0; 88.0	−92.9; -12.0
Mean reduction (%)			−66.8

Note: SALT 100 indicates complete absence of hair and SALT 0 indicates no hair loss on the scalp.

*t* test: ^a^ p = 0.003; ^b^ p < 0.001.

**Figure 1:**
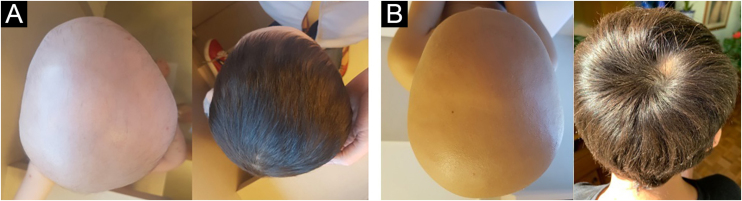
Clinical response to mesalazine in patients with universal alopecia areata. (A) Patient aged three years and six months, before and after 12 months of treatment. (B) Seven-year-old patient, before and after 12 months of treatment

**Table 2:** Patients with AA and their response to mesalazine therapy at six and 12 months

Patient number	Sex	Age at AA onset (months)	Age at the start of mesalazine therapy (years)	Mesalazine dosage (mg/kg/day)	AA subtype	SALT_B_	SALT_6M_ (% of reduction)	Response^1^	SALT_12M_ (% of reduction)	Response^1^
1	F	132	12	30	Plaques	40.6	62.6	Significant	100.0	Highly significant
2	F	60	7	35	Plaques	43.4	31.8	Moderate	48.6	Moderate
3	M	72	12	48	Plaques	71.6	27.9	Moderate	60.6	Significant
4	F	48	6	40	Plaques	91.0	24.3	Low	45.9	Moderate
5	M	84	8	39	UA	97.0	7.1	Low	95.8	Highly significant
6	F	36	14	30	TA	98.0	61.6	Significant	82.4	Highly significant
7	F	31	3	43	UA	98.0	59.2	Significant	73.5	Significant
8	M	60	7	30	TA	98.0	25.1	Low	65.9	Significant
9	M	72	3	47	UA	100.0	67.2	Significant	90.7	Highly significant
10	M	3	6	30	TA	100.0	11.8	Low	12.0	Low
11	M	36	14	40	UA	100.0	0	No response	‒	‒
12	M	60	9	35	UA	100.0	0	No response	‒	‒
13	F	72	14	30	TA	100.0	#	#	#	#
14	M	60	15	30	UA	100.0	#	#	#	#
15	F	21	9	30	Diffuse	*	*	Good	*	Excellent
16	F	36	4	37.5	Diffuse	*	*	Excellent	*	Excellent
17	F	72	14	40	Diffuse	*	*	Good	*	Excellent
18	F	12	9	30	Diffuse	*	*	Excellent	*	Excellent

Response scale in patients with diffuse AA: repilation > 40% (excellent); 20%‒40% (good); <10% (low).

1 Response scale in patients with plaque AA, UA or TA: -5% to -25% (low); -26% to -50%: moderate; -51% to -75%: significant; -76% to -100%: highly significant.

* In patients with diffuse AA subtype, SALT cannot be applied.

# Patients who did not follow treatment due to their moving to another city or due to the COVID-19 pandemic and could not be evaluated.

In 2007, a study showed that a mesalazine-like drug called sulfasalazine maintained corticosteroid-induced repilation, suggesting a potential benefit of this medication.^8^ Sulfasalazine is a prodrug consisting of 5-aminosalicylic acid (ASA) linked to sulfapyridine through an azole ring. While 5-ASA has beneficial effects in inflammatory bowel disease, sulfapyridine is responsible for most of the side effects (headache, anorexia, nausea and vomiting). Mesalazine (slow-release 5-ASA) does not contain sulfapyridine and is better tolerated. It is used in children for the treatment of inflammatory bowel disease and has immunomodulatory and immunosuppressive effects, including inhibition of interleukin (IL)-1 and IL-2 and tumor necrosis factor-alpha (TNF-α).^2^

In 2018, the authors group published the first trial with mesalazine and demonstrated its benefit in pediatric patients with severe AA.^2^ In 2021, Mahgoob et al. compared mesalazine with azathioprine in 30 adult and child patients with severe AA and showed that both are effective for AA, with mesalazine showing fewer side effects.^9^ Our study observed a mean relative SALT reduction of 66.8% after 12 months of treatment with mesalazine, a result similar to a study that showed a 67.7% reduction in adolescents with AA using tofacitinib;^10^ however, at a much lower cost of treatment in the case of mesalazine. The present study highlights the potential benefits of mesalazine for managing patients with AA. It seems to be a safe possibility, especially in the pediatric population, in which the use of drugs with a lower probability of side effects is imperative. Study limitations are the small sample size and the concomitant use of corticosteroid therapy when disease was active. More studies, especially controlled clinical trials, must be carried out.

## Financial support

None declared.

## Authors' contributions

Analú Vivian: Drafting and editing of the manuscript.

Vania Oliveira de Carvalho: Review of the literature and drafting of the manuscript.

Ana Elisa Kiszewski: Design and planning of the study; critical review of the manuscript and approval of the final version of the manuscript.

## Conflicts of interest

None declared.

## References

[1] Ramos P.M., Anzai A., Duque-Estrada B., Melo D.F., Sternberg F., Santos L.D.N. (2020). Consensus on the treatment of alopecia areata ‒ Brazilian Society of Dermatology. An Bras Dermatol.

[2] Kiszewski A.E., Bevilaqua M., Abreu L.B.D. (2018). Mesalazine in the treatment of extensive alopecia areata: a new therapeutic option?. Int J Trichology.

[3] Hamilton C.E., Craiglow B.G. (2020). JAK Inhibitors for the treatment of pediatric alopecia areata. J Investig Dermatol Symp Proc.

[4] Wohlmuth-Wieser I., Osei J.S., Norris D., Price V., Hordinsky M.K., Christiano A. (2018). Childhood alopecia areata ‒ data from the National Alopecia Areata Registry. Pediatr Dermatol.

[5] Nanda A., Al-Hasawi F., Alsaleh Q.A. (1999). A prospective survey of pediatric dermatology clinic patients in Kuwait: an analysis of 10,000 cases. Pediatr Dermatol.

[6] Olsen E.A., Hordinsky M.K., Price V.H., Roberts J.L., Shapiro J., Canfield D. (2004). Alopecia areata investigational assessment guidelines — Part II National Alopecia Areata Foundation. J Am Acad Dermatol.

[7] Bilgiç Ö, Bilgiç A., Bahalı K., Bahali A.G., Gürkan A., Yılmaz S. (2014). Psychiatric symptomatology and health-related quality of life in children and adolescents with alopecia areata. J Euro Acad Dermatol Venereol.

[8] Bakar O., Gurbuz O. (2007). Is there a role for sulfasalazine in the treatment of alopecia areata?. J Am Acad Dermatol.

[9] Mahgoob R.A.S., Elgamal E.E., Elshahat O.M., Almetwaly S.A. (2022). Comparative study between the efficacies of azathioprine and mesalazine in the treatment of severe alopecia areata. J Cosmet Dermatol.

[10] Jerjen R., Meah N., Carvalho LT de, Wall D., Eisman S., Sinclair R. (2021). Treatment of alopecia areata in pre-adolescent children with oral tofacitinib: a retrospective study. Pediatr Dermatol.

